# Supplementary material for: Effects of platelet-rich plasma on mesenchymal stem cells isolated from rat uterus
Source: PeerJ. 2020 Nov 30;8:e10415. doi: 10.7717/peerj.10415 (PMC7713597; doi:10.7717/peerj.10415)
Supplement: Supplemental Information 1 — Raw membranes after chemiluniniscence visualization [file peerj-08-10415-s001.zip › WB membranes/gapdh-2.pdf]

Image ID: 0000018_01
Acquire Time: 26.02.2020 15:27:41

Acquisition Information

#	Image ID	Acquire Time	Sensitivity	Image Name	Comment	Image Modifications	Project
1	0000018_01	26.02.2020 15:27:41	High	gapdh			

Image Display Values

Channel	Color	Minimum	Maximum	K
Chemi	Gray Scale (Black on White)	2,33	121	0
