# Supplementary figures and images for: Effects of platelet-rich plasma on mesenchymal stem cells isolated from rat uterus
Source: PeerJ. 2020 Nov 30;8:e10415. doi: 10.7717/peerj.10415 (PMC7713597; doi:10.7717/peerj.10415)

## Image Report: GAPDH

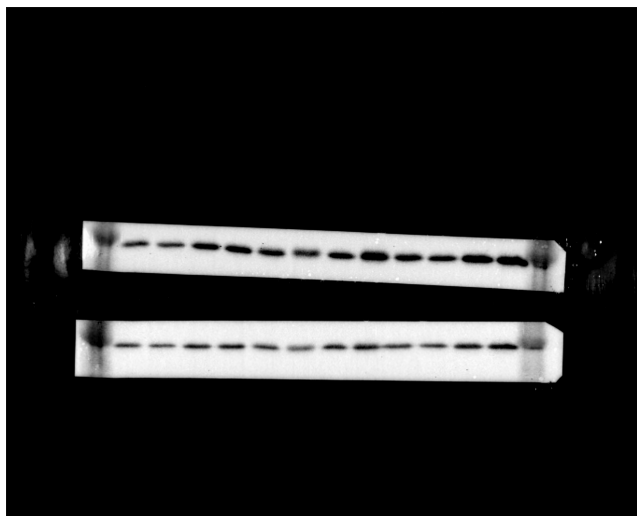

C:\Users\Полина\Desktop\Polina Vishnyakova\ChemiDoc Images  
2020-08-31\_11.51.56\GAPDH.scn

---

Supplement: Supplemental Information 1 — Raw membranes after chemiluniniscence visualization [file peerj-08-10415-s001.zip › WB membranes/GAPDH 3 4.pdf]

## Image Report: LC3B

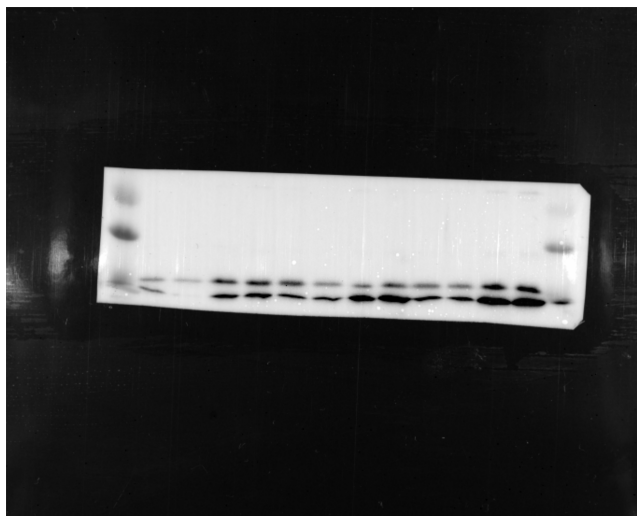

C:\Users\Полина\Desktop\Polina Vishnyakova\ChemiDoc Images 2020-08-31\_11.51.56\LC3B.scn

---

Supplement: Supplemental Information 1 — Raw membranes after chemiluniniscence visualization [file peerj-08-10415-s001.zip › WB membranes/LC3B.pdf]

## Image Report: MMP9

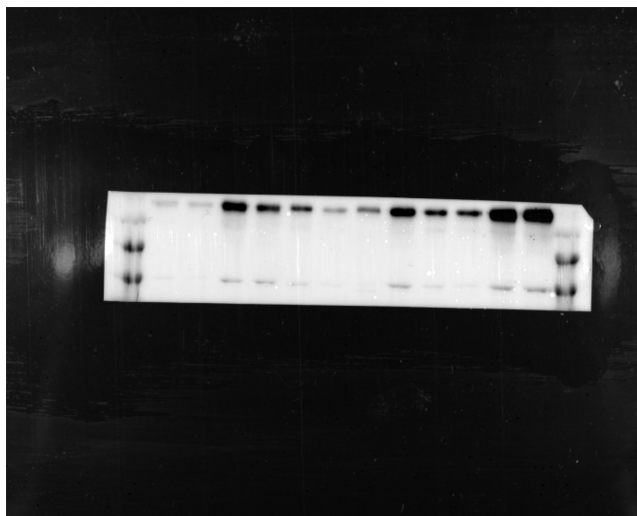

C:\Users\Полина\Desktop\Polina Vishnyakova\ChemiDoc Images 2020-08-31\_11.51.56\MMP9.scn

---

Supplement: Supplemental Information 1 — Raw membranes after chemiluniniscence visualization [file peerj-08-10415-s001.zip › WB membranes/MMP9.pdf]
