# Supplementary material for: Effects of platelet-rich plasma on mesenchymal stem cells isolated from rat uterus
Source: PeerJ. 2020 Nov 30;8:e10415. doi: 10.7717/peerj.10415 (PMC7713597; doi:10.7717/peerj.10415)
Supplement: Supplemental Information 3 [file peerj-08-10415-s003.pdf]

Supplementary information

Effects of platelet-rich plasma on mesenchymal stem cells isolated from rat uterus

Vishnyakova Polina, Artemova Daria, Elchaninov Andrey, Efendieva Zulfiia, Apolikhina Inna, Sukhikh Gennady and Fatkhudinov Timur

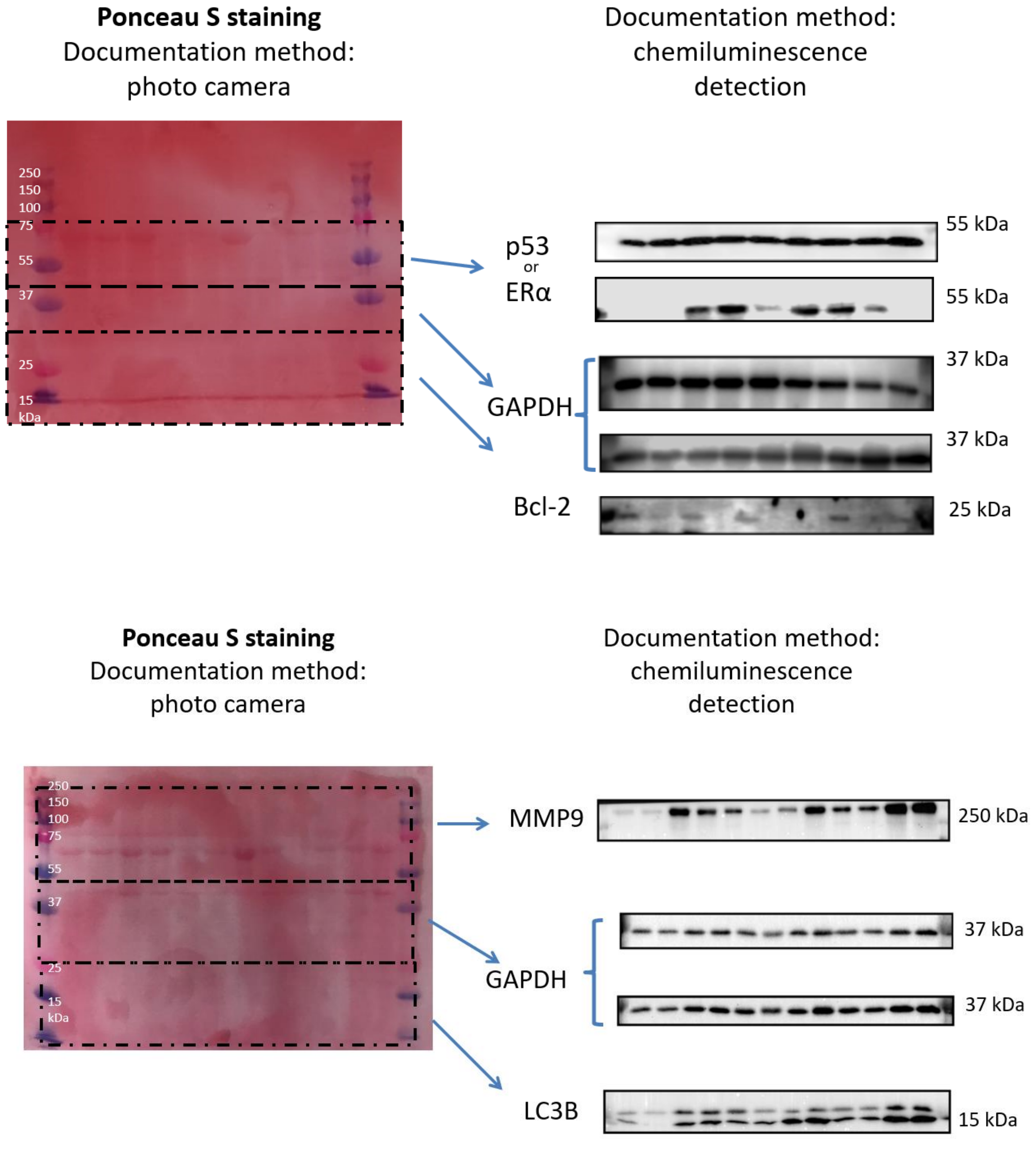

Fig.S1. Full-size membrane after blotting of polyacrylamide gel. Schemes of membrane Ponceau S staining. After visualization of the proteins with Ponceau S membranes were cut as indicated with a dotted line and stained with mentioned antibodies.
